# Recent Development on Plant Aldehyde Dehydrogenase Enzymes and Their Functions in Plant Development and Stress Signaling

**DOI:** 10.3390/genes12010051

**Published:** 2020-12-31

**Authors:** Adesola J. Tola, Amal Jaballi, Hugo Germain, Tagnon D. Missihoun

**Affiliations:** Groupe de Recherche en Biologie Végétale (GRBV), Department of Chemistry, Biochemistry and Physics, Université du Québec à Trois-Rivières, 3351 boul. des Forges, Trois-Rivières, QC G9A 5H7, Canada; adesola.tola@uqtr.ca (A.J.T.); Amal.Jaballi@uqtr.ca (A.J.); hugo.germain@uqtr.ca (H.G.)

**Keywords:** aldehyde dehydrogenases, abiotic stress, gene density, stress signaling, protein carbonylation

## Abstract

Abiotic and biotic stresses induce the formation of reactive oxygen species (ROS), which subsequently causes the excessive accumulation of aldehydes in cells. Stress-derived aldehydes are commonly designated as reactive electrophile species (RES) as a result of the presence of an electrophilic α, β-unsaturated carbonyl group. Aldehyde dehydrogenases (ALDHs) are NAD(P)^+^-dependent enzymes that metabolize a wide range of endogenous and exogenous aliphatic and aromatic aldehyde molecules by oxidizing them to their corresponding carboxylic acids. The ALDH enzymes are found in nearly all organisms, and plants contain fourteen ALDH protein families. In this review, we performed a critical analysis of the research reports over the last decade on plant ALDHs. Newly discovered roles for these enzymes in metabolism, signaling and development have been highlighted and discussed. We concluded with suggestions for future investigations to exploit the potential of these enzymes in biotechnology and to improve our current knowledge about these enzymes in gene signaling and plant development.

## 1. Introduction

Abiotic and biotic stresses increase the levels of the reactive oxygen species (ROS) including hydrogen peroxide, hydroxyl radical and superoxide radical in plants. Developing crops with the enhanced capability to maintain low ROS levels and reduce their damaging effects has remained an important goal in plant physiology, biotechnology and breeding. The superoxide anion radical and hydrogen peroxide can directly oxidize lipids or be converted to a hydroxyl radical in vivo through the Fenton and Haber-Weiss reactions. A hydroxyl radical readily initiates the peroxidation of the poly-unsaturated fatty acids (PUFAs), mainly linoleic and linolenic acids. Spontaneous rearrangements of the oxidized PUFAs lead to the generation of various phytoprostanes and aldehydes such as 4-hydroxy-2-nonenal, hexanal, (E) 2-hexenal and malondialdehyde (MDA) [1,2]. Many of the lipid peroxidation products, including MDA, are commonly designated as reactive electrophile species (RES) as they contain an electrophilic (electron-accepting) α, β-unsaturated carbonyl group. To cope with the detrimental effects of RES, the cells use diverse enzymatic and non-enzymatic detoxifying systems. Reactive aldehydes are detoxified by either the reduction of their carbonyl group to alcohol or the oxidation to the corresponding carboxylic acid [3]. The oxidative reaction is catalyzed by NAD(P)^+^-dependent aldehyde dehydrogenases (ALDHs, EC 1.2.1.3) that represent a large protein superfamily, of which, the members are widely distributed from human to plant genomes [4,5]. The *ALDH* genes are named according to the nomenclature proposed by Vasiliou et al. [4]. The gene names contain the root symbol “ALDH”, followed by a number representing the family and a letter representing the subfamily. The last number, after the letter of the subfamily, denotes the individual gene within that subfamily. Gene subfamilies and numbers are given chronologically following their identification. ALDH proteins that share at least 40% and 60% sequence identity are grouped into the same family and the same subfamily, respectively. Several human ALDH enzymes also catalyze ester hydrolysis [6]. The last twenty years have witnessed important insights in plant ALDH proteins [7,8,9,10,11,12] but our knowledge about their functions has significantly improved since the last review on the subject [13]. In this review article, we provided a detailed analysis of the research reports over the last decade with an emphasis on the roles of plant ALDHs in metabolism, signaling and development. We also highlighted the areas still obscure that require further research to understand the role of the plant ALDHs in the control of gene expression, metabolism, growth and development.

## 2. The ALDH Superfamily

Plants contain fourteen ALDH protein families—of which, the families ALDH11, ALDH12, ALDH19, ALH21, ALDH22, ALDH23 and ALDH24 are only found in plants, whereas the families ALDH2, ALDH3, ALDH5, ALDH6, ALDH7 and ALDH10 are also present in humans [13]. In comparison to Arabidopsis, which has 16 *ALDH* genes represented in 10 protein families, almost twice this gene number was found in species such as *Malus domestica* Borkh. (39 genes), *Gossypium raimondii* Ulbr. (30 genes), *Solanum lycopersicum* L. (29 genes)*, Brassica rapa subsp. pekinensis* (Lour.) Hanelt (27 genes) and *Cicer arietinum* L. (27 genes) [13,14,15,16,17,18]. The major driver of the ALDH family expansion is not clear so far. In contrast to the number of *ALDH* genes, the *ALDH* gene density, which we defined as the ratio of the genome size to the number of *ALDH*-coding genes in the genome, reflects well on how the expansion of the *ALDH* gene families followed that of the species genome (Table 1). However, no trend could be seen between the developmental complexity of the species (unicellular versus multicellular and vascular versus non-vascular) and the *ALDH* gene density or the number of *ALDH* genes in the species. This, apparently, supports the thesis that plants contain a core set of ALDHs to which new *ALDH* genes are added or lost during genome expansion [14]. However, further studies are required to better understand the distribution of the *ALDH* genes during evolution. Indeed, no information is currently available on ALDHs in streptophyte algae, which contain the closest algal relatives of land plants and are of great interest to understanding the evolutionary roots of stress responses in terrestrial plants [19]. A lack of knowledge is also noted about the ALDHs in many land plants, including ferns, *Marchantia polymorpha* L., 1753 and *Anthoceros* hornworts [20,21,22]. Moreover, it remains unclear what drove the change of the core set of ALDHs in the unicellular species and the multicellular vascular and non-vascular species. We hypothesize that environmental conditions and metabolic requirements might play a major role.

### 2.1. ALDH Catalytic Activities and Importance in Plant Development

The ALDH enzymes require pyridine cofactors for their catalytic activity. Most plant ALDHs tend to prefer NAD^+^ over NADP^+^, except the ALDH11, ALDH18 and ALDH19 family proteins that were shown to use NADP exclusively [15,16,17]. By using the Arabidopsis ALDH3H1 as an example, Stiti, et al. [18,43] showed that the residues E149, V178 and I200 are critical for the specificity toward NAD^+^. The site-directed mutagenesis of these amino acid residues into threonine, arginine and valine, respectively, shifted the cofactor preference to NADP^+^ [18]. Kinetic studies of a few plant and animal ALDHs showed that their catalytic mechanism follows a Bi-ordered steady-state kinetic [44,45]. Most ALDHs have a broad substrate specificity, meaning that they can oxidize a range of aliphatic aldehydes compounds, whereas few showed a narrow specificity. These include members of the subfamily ALDH2C and of the families ALDH5, ALDH6, ALDH10, ALDH11, ALDH12, ALDH18 and ALDH19 [9,13]. As a consequence of their broad substrate specificity and presence in nearly all cell compartments, the ALDHs are very redundant in their functions [11]. Stiti et al. [46] investigated the possibility of probing ALDH activity in vivo by active site-labeling. The labeling of the Arabidopsis ALDH3H1 with a chloroacetamide probe occurred at the catalytic Cys and correlated with the enzyme activity. However, the probe also targeted other ALDHs than ALDH3H1 and became inhibited by the enzyme co-factor NAD^+^. These observations illustrated the difficulty to relate the function of an ALDH isoform in the cell to its activity towards a substrate. Despite this apparent limitation, recent studies revealed the substrate specificity of a few enzymes of the subfamily ALDH2C and of the families ALDH3 and ALDH10 and the association with physiological processes.

#### 2.1.1. ALDH2

The ALDH2 enzymes are mitochondrial proteins, except for the subfamily 2C enzymes that are cytosolic [47,48]. Like most ALDH enzymes, the ALDH2 enzymes can oxidize a large number of aliphatic aldehyde substrates, but the subfamily 2C prefers aromatic substrates. The ALDH2C4 protein from *A. thaliana* was shown to oxidize sinapaldehyde and coniferaldehyde into sinapic acid and ferulic acid, respectively [47]. The findings indicated that these ALDHs are also involved in terpenoid metabolism in citrus [49]. Numerous compounds are derived from benzoic acid in plants, which include cytokinins, salicylic acid, glucosinolates and benzenoids, which constitute the aroma of many fruits and natural volatile compounds [50,51,52,53]. An ALDH2 enzyme in *Antirrhinum majus* L. (snapdragon) was shown to oxidize benzaldehyde into benzoic acid via the non-β-oxidative route in vivo [54]. Besides aliphatic and arylic aldehydes, pyrazole and imidazole containing aldehydes were shown to be oxidized by maize ALDH2C-type enzymes. The maize recombinant ALDH2C1 enzyme (RF2C) readily oxidized 1-pyrimidinyl-4-imidazole carbaldehyde, 3-pyrazole carbaldehyde, 5-pyrazole carbaldehyde and 1-benzyl-4-pyrazole carbaldehyde, while the recombinant ALDH2C4 enzyme efficiently oxidized 2-phenyl-4-imidazole carbaldehyde [55]. These azole-containing aldehyde compounds are often intermediates of alkaloids and synthetic drugs, suggesting that ALDH2C proteins may be involved in the biosynthesis of azole-containing pesticides or repellents produced by plants. These findings indicate that plant ALDH2C enzymes may serve to engineer the biosynthesis of floral scents and many plant natural compounds via the non-β-oxidative pathway of benzoic acid biosynthesis. The plant ALDH2C enzymes could also be used in synthetic biology for the production of imidazole and pyrazole-derivatives used as antibiotics or antifungal drugs. In this regard, the comparison of the ALDH2C amino acid sequence revealed that few amino acid residues are conserved in dicotyledons but substituted by non-equivalent residues in monocotyledons [56]. For instance, ALDH2C1 contains a phenylalanine at position 466, which is conserved in the ALDH2C isoforms in the monocots *Brachypodium distachyon* (L.) P.Beauv., *Oryza sativa* L., *Sorghum bicolor* (L.) Moench, and *Setaria italica* (L.) P.Beauv. However, the homologs of the *ALDH2C* gene in the dicots *A. thaliana*, *G. raimondii*, *Populus trichocarpa* Torr. & A.Gray, *Glycine max* (L.) Merr. and *Eutrema salsugineum* (Pall.) contain a tyrosine in the equivalent position. Protein structure modeling suggested that the presence of tyrosine would widen the substrate-binding pocket in the dicotyledons and thereby influence substrate specificity [56]. These findings remain to be verified experimentally, but they suggest that specific amino acid substitutions between the ALDH2C isoforms in dicotyledon and monocotyledon species could further fine-tune the enzyme activity toward secondary metabolite substrates.

Few studies have underlined the importance of the ALDH2 enzyme activity in plant development. Cui et al. [7] isolated the first ALDH gene from maize that encodes a mitochondrial class-2 ALDH (*Rf2a* or ALDH2B2). ALDH2B2 (*Rf2a*) was identified as the nuclear restorer of cytoplasmic male sterility in maize and shown to be involved in anthers development [7,48,57], but the mechanisms of action were unclear. Very recently, Xie et al. [58] showed that *OsALDH2b*, an orthologous gene of *ALDH2B2* in rice (*O. sativa*), negatively regulates tapetal-programmed cell death. *OsALDH2b* was highly expressed in anthers from meiosis to the early microspore stage. Plants deficient in *OsALDH2b* accumulated excess malondialdehyde and showed early programmed cell death in the tapetum, causing premature tapetum degeneration and abnormal microspore development [58]. These results provided further insight into the role of ALDH2B proteins in anthers development.

#### 2.1.2. ALDH3

Zeaxanthin is the common precursor for three major classes of apocarotenoids: crocins, picrocrocin and safranal. The cleavage of zeaxanthin by carotenoid cleavage dioxygenase 2 (CCD2) leads to 3-OH-β-cyclocitral and crocetin dialdehyde. Studies in saffron, a spice produced from the stigmas and styles of *Crocus sativus* L., suggested that ALDH3I1 is involved in the oxidation of crocetin dialdehyde to crocetin [59,60]. CsALDH3I1 was shown to have a strong preference to β-apo-8’-carotenal. This activity is reminiscent to that of ALDH3H1, which oxidizes ether-alkane aldehyde in the jojoba plant (*Simmondsia chinensis* (Link) C.K.Schneid.) [61]. So far, only the ALDH2C and ALDH3 enzymes were shown to efficiently oxidize aromatic and long-chain aldehydes. They could be useful in synthetic biology, as shown recently by Liu et al. [62].

#### 2.1.3. ALDH5, ALDH6 and ALDH7

The plant ALDH5 genes encode a succinic semialdehyde dehydrogenase involved in the operation of the GABA shunt pathway (see, also, ALDH21 below) [63,64]. The ALDH6 protein family is represented by a single gene, *AT2G14170,* encoding a methylmalonate-semialdehyde dehydrogenase in *A. thaliana* and in rice [9,11,13]. The enzyme is proposed to be involved in the oxidative decarboxylation of methylmalonate semialdehyde into propionyl-CoA during the catabolism of branched-chain amino acids [65]. The gene could be involved in the recycling of carbon skeletons from amino acid degradation during senescence or stress. The findings also suggested that the enzyme might play a role in root development and leaf sheath elongation in rice [66]. Mutant analyses are required to elucidate the physiological role of ALDH6B2 in plants. The plant ALDH7 enzymes oxidize a broad range of aldehyde substrates [67]. ALDH7 proteins appear to be expressed in all plant tissues and to be the most stress-inducible ALDH proteins [10,68,69]. The ALDH7B4 in *A. thaliana* was also induced upon herbicide treatment [70]. The mutation of the *ALDH7* gene in rice led to impaired seed development due to increasing Amadori products and reactive aldehydes [12]. Li et al. reported that a transgenic soybean line overexpressing an aldehyde dehydrogenase gene developed larger seeds than the wild-type genotype, but the identity of that gene was not properly indicated [71]. These findings collectively indicate the implication of the *ALDH* genes in seed development, size and viability.

#### 2.1.4. ALDH10

The plant ALDH10 enzymes are aminoaldehyde dehydrogenases (AMADHs, EC 1.2.1.19) involved in the oxidation of several nitrogen-containing aldehydes, including betaine aldehyde. They are associated with polyamine catabolism and the biosynthesis of compatible solutes, fragrance and carnitine [11,72,73,74,75]. AMADH1 in tomatoes (*S. lycopersicum*) was also used to detect furfural and its derivatives as side-products of the thermal degradation of sugars in plum brandy [76]. These findings underline the potential of these enzymes in plant biotechnology. Biochemical analyses showed that the high catalytic efficiency of AMADHs towards ω-aminoaldehydes is defined by the presence of aspartic acid and aromatic residues in the substrate channel [77,78]. Studies in peas (*Pisum sativum* L.) found that substrates with diverse branching and acyl chain lengths derived from the natural aminoaldehyde substrate had less influence on the enzyme activity than the size of the natural non-branched aminoaldehyde substrates [79]. Moreover, the oxidation of betaine aldehyde by the ALDH10 enzymes generate glycine betaine (GB), which accumulates in the plant cells as an osmolyte under osmotic stress. However, not all plants accumulate GB, although most plants express two functional *ALDH10* genes [73,80,81,82]. For example, spinach (*Spinacia oleracea* L.) is a GB accumulator, whereas *A. thaliana* is not. The analysis of the crystal structure of spinach betaine aldehyde dehydrogenase (SoBADH) uncovered what confers BADH activity to the ALDH10 enzymes. Diaz-Sanchez et al. [83] showed that tyrosine Y160 and tryptophan W456 (spinach enzyme numbering) are strictly conserved in plant ALDH10s and form a pocket that accommodates the bulky trimethylammonium group. This pocket is reduced in ALDH10s with low BADH activity, because an isoleucine pushes tryptophan against tyrosine. In comparison, ALDH10s with high BADH activity have alanine (A441 in SoBADH) or cysteine instead of isoleucine, which leaves enough room for the binding of BAL. Accordingly, the mutation A441I decreased the catalytic efficiency of SoBADH about 200 times, while the mutation A441C had no effect [83]. Phylogenetic analyses suggested that the BADHs with high catalytic efficiency evolved from ancestral AMADHs through the mutations I441A or I441C (SoBADH numbering) [84]. The overexpression of ALDH10 isoforms with high BADH activity was investigated in several studies and showed to confer stress tolerance to the plants. Recently, the two ALDH10 proteins in *A. thaliana*, AtALDH10A8 and AtALDH10A9, were shown to oxidize trimethylaminobutyraldehyde into γ-butyrobetaine, the precursor of carnitine [85]. The double mutants contained low γ-butyrobetaine level, but no difference was found in the carnitine contents of the wild type and the mutants. While the single mutant had no phenotype under normal growth conditions, a high number of seeds failed to mature in the siliques of the double mutants, and the seeds that matured germinated faster than the wild type. The Arabidopsis ALDH10 enzymes thus appeared to be involved in seed development via the synthesis of γ-butyrobetaine. The precise function of γ-butyrobetaine in seed development remains to be elucidated.

#### 2.1.5. ALDH11

ALDH11 encodes non-phosphorylating glyceraldehyde-3-phosphate dehydrogenase (GAPN; E.C. 1.2.1.9), which oxidizes glyceraldehyde-3-phosphate to 3-phosphoglycerate in a NADP^+^-dependent manner in the cytosol [86]. The enzyme acts as a vehicle for NADPH export from the chloroplast to the cytosol. There is one *ALDH11* gene in *A. thaliana*, and a loss-of-function mutation in ALDH11 altered the expression of enzymes of the carbohydrate metabolism and caused the repression of photosynthetic genes [87]. This underlines the importance of ALDH11 enzymes in the plant metabolism. The reaction catalyzed by ALDH11 parallels the oxidative phosphorylation of glyceraldehyde-3-phosphate to 1,3-bisphosphoglycerate by phosphorylating glyceraldehyde-3-phosphate dehydrogenases (GAPDHs, E.C. 1.2.1.12). By competing for the same substrates with GAPDHs, the ALDH11 enzyme likely acts as a node of regulation for plant glycolysis.

#### 2.1.6. ALDH12, ALDH18 and ALDH19

Proline was also shown to function in seed germination, root elongation, flowering, embryo development and pollen fertility [88,89,90,91,92,93,94]. The ALDH12 proteins encode a NAD^+^-dependent glutamate γ-semialdehyde dehydrogenase (GSALDH) that oxidizes glutamate γ-semialdehyde (GSAL), a mitochondrial intermediate of the proline and arginine catabolism, to glutamate. As proline catabolism enzymes, they were shown to be downregulated under high salt or dehydration stresses in maize and the moss *P. patens* to allow the accumulation of proline as a compatible solute [95]. They are involved in proline homeostasis. In contrast to ALDH12, the ALDH19 family codes for the γ-glutamyl phosphate reductase involved in proline biosynthesis. The only member known for this family was found in tomatoes [96,97]. The γ-glutamyl phosphate reductase enzyme was found as a bifunctional protein showing also γ-glutamyl kinase (GK) activity [96]. Using the entire sequence of this bifunctional enzyme in the blast searches may not give significant hits because of the coding region for γ-glutamyl kinase activity. However, using solely the sequence of the γ-glutamyl phosphate reductase may help find other potential ALDH19 proteins in other species. Similarly, the plant *{Delta} 1-Pyrroline–5-Carboxylate Synthetase (P5CS)*, encoded by a gene of the ALDH18 protein family, is involved in the biosynthesis of proline. The P5CS protein is a bifunctional enzyme that has amino-acid kinase and aldehyde dehydrogenase activities. Proline is an osmoprotectant that accumulates in plants under stress conditions. The ability of the plants to accumulate important levels of proline to fight against stress caused by low water potential appeared to evolve with time [98]. Genetic variations in proline accumulation and the associated stress tolerance were found between accessions of *A. thaliana* [99]. Downregulation of the *ALDH18* genes impaired the seed germination in *A. thaliana*, reduced the size of the root meristem and delayed bolting [93,100,101]. In contrast, the ectopic expression of ALDH18 in switchgrass (*Panicum virgatum* L., 1753) resulted in faster growth and earlier flower development than in wild-type plants [102]. Similarly, transgenic plants of *A. thaliana* overexpressing ALDH18 had a shorter vegetative growth in short-day conditions [103]. As the key proline biosynthetic genes, *ALDH18* genes appears to influence plant stress responses, vegetative growth and flowering. It remains unclear whether they are targeted by the same or different regulators in these physiological processes. The elucidation of this question may shed light on the question of whether normal flowering and stress-induced flowering are controlled by common mechanisms.

#### 2.1.7. ALDH21, ALDH22, ALDH23 and ALDH24

ALDH21 family proteins are absent in the flowering plants but are found in lower plants, including green algae, bryophytes, lycophytes and ferns. Bryophytes are non-vascular plants encompassing mosses, hornworts and liverworts. The ALDH21 protein family is represented by a single gene in *P. patens*, *S. moellindorffii*, *Syntrichia ruralis* Hedw. and *S. caninervis* [13,14,104,105,106]. The biochemical characterization of ALDH21 from *P. patens* revealed that it encodes a tetrameric NADP^+^-dependent succinic semialdehyde dehydrogenase (SSALDH) that converts succinic semialdehyde, an intermediate of the γ-aminobutyric acid (GABA) shunt pathway, into succinate in the cytosol [106]. The presence of a succinic semialdehyde dehydrogenase suggests the operation of the GABA shunt pathway in non-flowering species. The equivalent enzyme in the flowering plant *A. thaliana* is a mitochondrial protein of the ALDH5 family that uses NAD^+^ as a cofactor. Interestingly, both ALDH5 and ALDH21 are present in some lower plants, indicating that the *ALDH21* genes have been lost during the evolution of lower plant species to flowering species. This might bear some advantage for the control of the GABA shunt pathway in flowering species, since any disturbance of this pathway was shown to severely impair the growth and flower development in *A. thaliana* [63,107]. Functional studies showed that the *ALDH21* gene is induced by water deprivation in the mosses *S. ruralis* and *S. caninervis* [104,105] and its ectopic expression in tobacco and cotton led to drought and salt-tolerant plants [108,109]. As for ALDH22, ALDH23 and ALDH24, there has been no new reports on their activity and roles since the review work done by Brocker et al. [13]. Therefore, the readers are invited to consult this publication that summarized well the so-far available literature on these plant ALDH protein families.

### 2.2. ALDH Gene Regulation 

Gene expressions can be regulated at the transcriptional and posttranscriptional levels in eukaryotes. Previous findings showed that stress-inducible ALDHs are regulated at the transcriptional level by ABA [11,110]. Comparisons of the *ALDH7* gene promoter sequences of different Brassicaceae identified a conserved ACGT-containing motif and a dehydration-responsive element/C-repeat low temperature-responsive element, which control *ALDH7B4* expression in seeds and induction by salt, dehydration and ABA in leaves [68]. NAC (no apical meristem, NAM, ATAF1/2 and CUC2) transcription factors control diverse biological processes in response to stress, developmental and hormonal signals [111,112]. Several NAC transcription factors, including ATAF1, were found to bind and activate the *ALDH7B4* promoter [113]. ATAF1 directly binds to and activates the promoter. The overexpression of *ATAF1* (At1g01720) in Arabidopsis plants results in the elevated expression of *ALDH7B4* in seeds, seedlings and mature plants, whereas *ATAF1* knock-out mutant plants display abolished expressions of *ALDH7B4*. These findings indicate that *ALDH7B4* and likely other stress-responsive ALDHs that also contain ACGT and DRE/CRT motifs are common targets of NAC transcription factors. As in animals, alternative gene splicing has been reported for a few plant *ALDH* genes. These include *ALDH3H1* in *A. thaliana* and *ALDH2B4* in grapevines [114,115]. For *ALDH3H1*, one variant lacks the first exon of the main transcript but contains a cryptic exon that is absent in the main gene transcript. The other *AtALDH3H1* splice variants contain premature stop codon leading to truncated proteins, but gene expression analyses revealed that some of these variants were induced upon water stress. Moreover, it was found that the gene contains a long first intron that harbors cis-elements that restrict the expression of the splicing variants to the base of fresh leaves near the shoot meristem location [114]. Likewise, in grapes, three splicing variants were identified: *VvALDH2B4*_v1, *VvALDH2B4*_v2 and *VvALDH2B4*_v3. *VvALDH2B4*_v1 and *VvALDH2B4*_v3 bear different 3′ splicing acceptor sites in the third exon, while *VvALDH2B*_v2 has an intron retention that leads to a different translation initiation site. The functions of these transcript variants in the two species remain unclear. The transcription of certain plant *ALDH* genes may also be controlled via intragenic methylation [116]. The methylation-sensitive amplification polymorphism (MSAP) assay revealed that *ALDH2B7a* in potatoes (*S. tuberosum*) is differentially methylated at cytosine sites within an intron and an exon in response to lower temperature treatments. Although several plant ALDHs are considered to be involved in the detoxification of stress-derived aldehydes, these findings suggest that some ALDHs with potent roles in primary and secondary metabolisms might be temporally repressed by this mechanism in other to avoid a waste of resources under stress conditions. In addition to the regulation at the transcriptional level, ALDHs can also be targeted by post-translational modifications. Cysteine in the catalytic site of ALDH3I1 and ALDH3H1 can undergo reversible S-nitrosylation in vitro, indicating a possible inactivation of these enzymes in vivo by nitrosylation [117]. Likewise, profiling of the carbonylated proteome in *A. thaliana* identified carbonylated ALDH7B4-derived peptides [118].

## 3. ALDH Roles in Abiotic Stress Responses

The enzymatic scavenging of aldehydes derived from stress-related lipid peroxidation involves ALDHs. The suppression of the *ALDH2C4* gene in *Nicotiana benthamiana* Domin led to plants that were more sensitive to lower-temperature stresses and accumulated more ROS and malondialdehyde [116]. The ALDH2B7a enzyme enhanced the cold stress tolerance in potatoes (*S. tuberosum*) [116]. The ectopic expression of the *AtALDH2B7* gene in *A. thaliana* under control of the drought-inducible promoter of the tryptophan-rich sensory protein (*TSPO*) gene also conferred drought stress tolerance to the transgenic plants [119]. Transgenic tobacco (*Nicotiana tabacum* L.) and *Arabidopsis* plants constitutively expressing the soybean *ALDH7* gene (*GmTP55)* were tolerant to high salinity during germination and to water deficit during plant growth [120]. These transgenic plants also exhibited an enhanced tolerance to oxidative stress by maintaining a low level of lipid peroxidation-derived aldehydes. Likewise, transgenic tobacco plants overexpressing the *ALDH22A1* gene from maize (*Zea mays* L.) showed increased stress tolerance accompanied by a reduction of MDA derived from the lipid peroxidation [121]. Three homologs of the *ALDH7B4* gene were found to be induced by osmotic stress in wheat [122]. The heterologous overexpression of the *TraeALDH7B1-5A* gene (present in chromosome 5A) in *A. thaliana* led to enhanced drought tolerance in transgenic plants [122]. Similarly, the overexpression of the *Brassica rapa* L., 1753 BrALDH7B2 protein in tobacco conferred a tolerance to salt and drought to seedlings [123]. The putative homologous gene of the *ALDH7* gene in the streptophyte alga *Mougeotia* was upregulated upon heat [124]. In *Arabidopsis*, *ALDH3I1* and *ALDH7B4* are strongly induced by dehydration, high salinity and heat stress, and the double mutant *aldh3i1 aldh7b4* was more sensitive to heat stress and stress combinations than wild-type plants [69,110]. Transgenic plants overexpressing both ALDH proteins were more tolerant to drought and salt stress than the wild type [10,125]. Unlike the wild-type plants, these transgenic plants could survive on media supplemented with 20-mM hydrogen peroxide. Collectively, these findings indicate that plant *ALDH7* and *ALDH3I* genes play an important role in stress tolerance in both dicotyledon and monocotyledon species. In comparison to *ALDH3I1*, the *ALDH3H1* gene was found to be upregulated by salt in seedling roots prominently [11], but the ALDH3H1 overexpressors did not perform better than wild-type plants under drought or high salinity conditions. Sub-cellular localization experiments revealed that ALDH3H1 is targeted to the cytosol, whereas ALDH3I1 contains a plastid transit signal that directs it to the chloroplasts. This implies that these proteins, although from the same family, could be functionally different. It is plausible that the ALDH3H1 protein, instead of having a primordial role in stress tolerance acquisition, could be involved in plant metabolism (see the section on catalytic activities) or maintenance of the root architecture and the integrity of root tissues under stress conditions. During the oxidation reaction catalyzed by the ALDHs, the protons derived from the aldehyde substrates are transferred to the cofactor NAD(P)^+^ to produce NAD(P)H. Reduced pyridine nucleotides are important cofactors for the ROS-detoxifying enzymes and for the turnover of oxidized glutathione and ascorbate [126]. The NAD(P)H/NAD(P) ratio in the chloroplasts or the mitochondria was shown to influence ROS homeostasis [127,128,129,130]. It was recently found that the mutation of the two prominent stress-inducible *ALDH* genes in *A. thaliana*, *ALDH3I1* and *ALDH7B4*, caused a decrease of the NAD(P)H and glutathione pools and lowered the NAD(P)H/NAD(P) ratio [131]. The double mutant *aldh3i1 aldh7b4* was also impaired in the xanthophyll cycle involved in the nonphotochemical quenching (NPQ) mechanism of the photosystem II. These observations indicated a shortage for reduced pyridine nucleotides and, together with the high levels of lipid peroxidation-derived aldehydes, pointed to a role of ALDHs as major contributors to the homeostasis of pyridine nucleotides in plants [131,132]. NAD(P)H generated by the activity of stress-induced ALDHs may be used for maintaining glutathione homeostasis and to support the functioning of ROS-detoxifying enzymes, such as the 2-alkenal reductases (AERs) and aldo-keto reductases (AKRs). Indeed, AERs and AKRs require NADPH to reduce the α, β-unsaturated bond and the carbonyl group of lipid-peroxidation-derived aldehydes to n-alkanal and alcohols, respectively [133,134,135]. In plant cells, the enzymes NADP-dependent isocitrate dehydrogenase (NADP-ICDH), glucose 6-phosphate dehydrogenase (G6PDH) and non-phosphorylating glyceraldehyde-3-phosphate dehydrogenase (GAPN) and NADP-malic enzyme2 (NADP-ME2) contribute to the NAD(P)H content. It will be interesting to examine the relative importance and the functional overlap between these enzymes and the ALDHs in the production of NAD(P)H for ROS detoxification under stress conditions.

## 4. ALDH Roles in Biotic Stress Responses

Records of the direct implication of ALDHs in the plant responses to pathogens are limited, but a few recent studies pointed out the contribution of plant ALDHs to the plant defense response against pests and pathogens. A proteomic approach was used to investigate the molecular pathways involved in the response of oak (*Quercus robur* L.) to three gall-inducing cynipid wasps *(Cynips quercusfolii, Cynips longiventris and Neuroterus quercusbaccarum)* [136]. A high level of ALDH2 proteins were associated with the interaction of the wasp *C. longiventris* with the host plant. The etiology of the increase in ALDH2 in the compatible interaction was unclear but could be to facilitate the nutrition of wasp larvae in the gall. Saini et al. [137] showed that yeast extract induced the accumulation of benzoic acid-derived phytoalexins aucuparin and noraucuparin in Asian pears (*Pyrus pyrifolia* (Burm.f.) Nakai, 1926). A benzaldehyde dehydrogenase and biphenyl synthase activity were concomitantly induced in the cells of *P. pyrifolia* treated by yeast extract, indicating an implication of benzaldehyde dehydrogenase in the biosynthesis of biphenyl phytoalexins. The gene responsible for the enzyme activity was not characterized, but a benzaldehyde dehydrogenase found in the petals of snapdragons (*A. majus*) was shown to derive from a protein with more than 80% sequence similarity with the plant ALDH2 family proteins [54]. It is, therefore, possible that plant ALDH2 enzymes are involved in the biosynthesis of benzoic acid-derived phytoalexins in response to elicitors and pathogen attacks. The ALDH gene expression was assessed during the interaction of *Plasmopara viticola* with grapevines [115]. *VvALDH11B1* was upregulated in both resistant and partially resistant lines but not in the susceptible line. In parallel, *VvALDH2B4*, *VvALDH2B9*, *VvALDH7B5* and *VvALDH10A9* were downregulated in the resistant lines, but no change was seen in the expression of these genes in the susceptible lines. The screening of transcriptomics datasets also revealed that several *ALDHs* genes were differentially expressed in grapevines after infection with the obligate biotrophic fungus *Uncinula necator* (Schw.) Burr. that causes powdery mildew, the leaf roll-associated closeter-ovirus-3 (*GLRaV-3*) and, during infection, by the *Bois Noir phytoplasma* [115]. Likewise, an ALDH gene, *CaALDH1*, in peppers (*Capsicum annuum* L., 1753) was strongly induced by avirulent *Xanthomonas campestris* pv. *vesicatoria* (Xcv) Ds1 (avrBsT) infection. The transient co-expression of CaALDH1 with avrBsT significantly enhanced avrBsT-triggered cell death in *N. benthamiana* leaves. Bimolecular fluorescence complementation and coimmunoprecipitation assays showed that CaALDH1 interacts with *Xanthomonas* type III effector AvrBsT to promote cell death in *N. benthamiana*. Ectopic overexpression of the *CaALDH1* gene in *A. thaliana* enhanced the defense response to *Pseudomonas syringae* pv. *tomato* and *Hyaloperonospora arabidopsidis* infections [138].

The focus of this review is primarily on the plant ALDH proteins. However, it is worth noticing the recent observations that downregulating the expression of some *ALDH* genes in plant pathogens negatively impacted their virulence. For example, the silencing of two putative family-four aldehyde dehydrogenase genes potassium-activated aldehyde dehydrogenase (*MoKDCDH*) and delta-1-pyrrorine-5-carboxylate dehydrogenase (*MoP5CDH*) in the rice blast pathogen *Magnaporthe oryzae* significantly compromised the pathogenesis of the fungus [139]. The silencing of *MoKDCDH* appeared sublethal in the mutants, thereby offering the possibility to target this enzyme for selective inhibition, since most plants do not express ALDH4 family proteins. Similarly, Norvienyeku, et al. [140] showed that methylmalonate-semialdehyde dehydrogenase of the rice blast pathogen *Magnaporthe oryzae* promotes the pathogenesis of the fungus by regulating the mobilization of Spitzenkörper during germ tube morphogenesis and the formation of appressoria by regulating small branch-chain amino acids, inositol, pyridoxine and AMP/cAMP homeostasis. These suggest that plant pathogens use their own ALDH to overcome the downregulation of the host plant ALDH enzymes as a virulence mechanism. Likewise, *Pseudomonas syringae* strain PtoDC3000 employs an indole-3-acetaldehyde dehydrogenase (*AldA*) to produce auxin indole-3-acetic acid to escape the host defense [141]. The *AldC* gene, homologous to *AldA,* was shown to function as a long-chain aliphatic aldehyde dehydrogenase, which likely contributed to the feeding of the pathogen on the aliphatic compounds in the plant apoplast [142,143,144]. Comparative and evolutionary studies of ALDH between plant and fungal or bacterial plant pathogens may help identify lineage-specific inhibitors to target the plant pathogen enzyme and control plant disease.

Altogether, much less is known about the role of *ALDH* genes in plant-microbe interactions when compared to the literature on the contribution of ALDHs to plant responses to abiotic stresses. While the ALDH2C proteins are likely involved in the biosynthesis of benzoic acid and benzoic acid-derived phytoalexins, the real effects of the differential expressions of other ALDH proteins remain to be investigated. ROS production is a well-known defense strategy deployed by plants to counter-attack pathogen infections. For instance, the hypersensitive response characterized by localized cell death is orchestrated by ROS produced by the plant during a compatible interaction. Recently, a new form of cell death, called ferroptosis, has been evidenced in plants. Ferroptosis depends on ROS-derived reactive lipid peroxides, but, currently, it is unclear how plants regulate ferroptosis [145,146,147,148]. We speculate that this may require the localized expression of ALDHs with a broad substrate specificity to circumvent cell death at the site of infection.

## 5. Recent Findings and Perspectives on the Role of Plant ALDHs in Gene Signalling

Besides their roles in the metabolism and the response to biotic and abiotic stresses, recent studies pointed to the role of ALDH2 genes in the regulation of plant developments and responses to stress. ALDH2B7 was shown as an enzyme of the pyruvate dehydrogenase bypass [149]. In this bypass, pyruvate decarboxylase enzymes convert pyruvate to acetaldehyde, which is then oxidized to acetate. Acetate may subsequently enter the tricarboxylic acid cycle or be used by acetylating enzymes. Interestingly, Arabidopsis histone deacetylase 6 (HDA6) was shown to repress the expression of pyruvate decarboxylase, and *ALDH2B7* genes and HDA6 mutants are tolerant to drought [150]. It appears that acetate produced by ALDH2B7 via the pyruvate dehydrogenase bypass serves for the acetylation of histones by histone acetyltransferase enzymes and promotes gene expression under stress conditions [150]. In mammalian cells, functional pyruvate decarboxylase can translocate, upon growth hormone or mitochondrial stress-derived signals, from the mitochondria to the nucleus during cell cycle progression to generate a nuclear pool of acetyl-CoA from pyruvate and increase the acetylation of core histones important for S phase entry [151]. Although the plant PDC1 was shown to localize in the cytosol [119], a possible translocation from the cytosol into the nucleus has not been accessed yet. The acetate derived from the ALDH enzymes in the cytosol may well translocate to the nucleus and serve for histone acetylation [152]. FLOWERING LOCUS C (FLC) is a master repressor of the flowering time. FLC represses SUPRESSOR OF OVEREXPRESSION OF CONSTANS1 (SOC1) and FLOWERING LOCUS T (FT), which induce the expression of genes required for the transition to flowering [153]. FLC expression is modulated by H3K9Ac and H3K14Ac and inhibited through H3K27me3 modification in response to cold stress [154,155]. Recently, Xu et al. [156] found that ALDH3F1 is involved in H3K9 acetylation on the FLC locus via acetate production. The mutation of *ALDH3F1* triggered early flowering, whereas the overexpression of ALDH3F1 caused late flowering. Considering the broad substrate specificity of ALDHs, these findings suggest that certain ALDH3F1 and ALDH2B enzymes could provide acetate to histone-acetylating enzymes, thereby connecting plant metabolisms and stress responses to the regulation of gene expressions for growth and development. More research is needed to clarify this hypothesis further. An investigation of the *A. thaliana* transgenic lines expressing the *ALDH7B4* gene promoter fused to the β-glucuronidase reporter gene showed that both pentanal and trans-2-hexenal induced the *ALDH7B4* gene expression. This suggested that proteins involved in the control of *ALDH* expression could be targeted to carbonylation by aldehydes [157,158]. *A. thaliana* contains three atypical extra-large G proteins (XLG1-3), in addition to the three canonical alpha, beta and gamma subunits of the G protein-coupled receptors (GPCR) [159,160]. The ALDH3H1 protein was shown to interact with the XLG protein in a yeast two-hybrid screening [161]. The interaction of ALDH3H1 with XLG may serve to retain XLG in the cytosol or protect it from covalent modification by the reactive aldehydes [157]. Protein carbonylation often leads to the degradation of targeted proteins and, therefore, may interfere with the cellular signaling pathways [162]. In agreement with this, ROS-mediated protein carbonylation has recently been demonstrated to mediate gene expression and signaling by ABA and auxins [163,164,165]. Future research should focus on identifying the signal transduction proteins targeted by carbonylation via the aldehydes and reactive electrophile species. Since auxins and ABA regulate several developmental processes in plants, the roles of ALDHs in fine-tuning the action of these phytohormones will also be useful.

## 6. Conclusions and Perspectives

The data presented here contributed to the understanding of the function and regulation of aldehyde dehydrogenases in plants over the last decade. Novel functions were identified for the ALDHs of the subfamilies 2B and 2C and families 3, 7 and 10. The ALDH2B proteins are involved in histone acetylation by generating acetate from acetaldehyde. They could, thereby, influence several developmental processes, including the development of floral organs. In addition to the previously known role of ALDH2C in the biosynthesis of sinapic acid and ferulic acid in *A. thaliana*, it appears that these proteins play a role in the plant response to pathogens through the biosynthesis of benzaldehyde-derived phytoalexins. Plant ALDH2C enzymes may be used to engineer floral scents and many plant natural compounds via the non-β-oxidative pathway of benzoic acid biosynthesis. The plant ALDH2C enzymes could also be used in synthetic biology for the production of imidazole and pyrazole derivatives used as antibiotics or antifungal drugs. Moreover, it would be interesting to investigate their role in the development and integrity of the plant cell wall. In the ALDH3 family, further studies are needed to understand the precise functions of the members. We suspect that gene neofunctionalization might diversify their function and the range of their preferred substrates in various species. A feature of the *ALDH3H1* gene uncovered in *A. thaliana* is the existence of an alternative promoter within the upstream intron that directs the expression of an alternative first exon transcript and several splicing variants. Further experiments are required to deepen the functional relevance of the *ALDH3H1* transcript variants. Findings have shown that ALDH3I1 and ALDH7B4 proteins are strongly responsive to stress and contribute to the availability of reducing equivalents for ROS detoxification [10,125,131]. Further studies are needed to examine the overlap between these ALDHs and other enzymes known to generate NAD(P)H for ROS detoxification or metabolism. New examples of the implication of the ALDH10 enzymes in the development of plant aromas were described in this review. This data underlines the potential of using these enzymes as a target to engineer aromas in plants. Lastly, the ALDHs, by oxidizing reactive carbonyl species, counteract protein carbonylation. Like protein ubiquitination, the carbonylation of the proteins leads to their degradation by the proteasome system. However, unlike the enzymatic control of the ubiquitination by deubiquitinases, protein carbonylation is a nonenzymatic and irreversible post-translational modification. Similar to deubiquitinase enzymes, ALDHs could also be involved in counteracting the deactivation of specific signal transduction proteins by carbonylation. We advocate that future research should include these areas to advance our knowledge about the plant ALDH functions beyond stress and in gene signaling and plant development.

## Figures and Tables

**Table 1:** Evolution of the *ALDH* gene number and *ALDH* gene density in selected plant species.

Clades	Species	Genome Size (Mbp)	*ALDH* Gene Number	^a^*ALDH* Gene Density (Mbp/ALDH)	^b^ References
Microalgae	*Ostreococcus tauri*	13	6	2.17	[23]
	*Volvox cateri*	138	7	19.71	[24]
	*Chlamydomonas reinhardtii*	118	9	13.11	[25]
Mosses	*Syntrichia caninervis*	45	15	3.03	[26]
	*Physcomitrella patens*	472	21	22.47	[27]
Vascular plants	*Arabidopsis thaliana*	136	16	8.47	[28]
	*Eutrema salsugineum*	243	17	14.30	[29]
	*Sorghum bicolor*	730	19	38.42	[30]
	*Oryza sativa*	372	20	18.60	[31,32]
	*Setaria italica*	423	20	21.15	[33]
	*Zea mays*	2106	22	95.73	[34]
	*Selaginella moellendorffii*	213	24	8.86	[35]
	*Vitis vinifera*	486	25	19.45	[36]
	*Populus trichocarpa*	423	26	16.27	[37]
	*Brassica rapa*	353	27	13.08	[38]
	*Solanum lycopersicum*	900	29	31.03	[39]
	*Gossypium raimondii*	748	30	24.93	[40]
	*Malus domestica*	650	39	16.66	[41]
	*Glycine max*	1013	53	19.12	[42]

^a^ The *ALDH* gene density represents the ratio of the genome size to the number of aldehyde dehy-drogenase (ALDH)-coding genes in the genome. ^b^ The genome size information was retrieved from the database Ensembl for plants, https://plants.ensembl.org/, if not otherwise stated.

## Data Availability

No new data were created in this study. Data sharing is not applicable to this article.
